# Assessment and Therapeutic Modulation of Heart Rate Variability: Potential Implications in Patients with COVID-19

**DOI:** 10.3390/jcdd10070297

**Published:** 2023-07-12

**Authors:** Luiz Schnekenberg, Annahita Sedghi, Daniela Schoene, Lars-Peder Pallesen, Jessica Barlinn, Felix Woitek, Axel Linke, Volker Puetz, Kristian Barlinn, Norman Mangner, Timo Siepmann

**Affiliations:** 1 Department of Neurology, Faculty of Medicine Carl Gustav Carus, Technische Universität Dresden, 01307 Dresden, Germany; luiz.schnekenberg@ukdd.de (L.S.); annahita.sedghi@ukdd.de (A.S.); daniela.schoene@ukdd.de (D.S.); lars-peder.pallesen@ukdd.de (L.-P.P.); jessica.barlinn@ukdd.de (J.B.); volker.puetz@ukdd.de (V.P.); kristian.barlinn@ukdd.de (K.B.); 2 Dresden Heart Center, Medical Faculty Carl Gustav Carus, Technische Universität Dresden, 01307 Dresden, Germany; felix.woitek@herzzentrum-dresden.com (F.W.); axel.linke@herzzentrum-dresden.com (A.L.); norman.mangner@herzzentrum-dresden.com (N.M.)

**Keywords:** SARS-CoV-2, COVID-19, heart rate variability, cardiac, cardiovascular, parasympathetic, sympathetic, autonomic

## Abstract

Cardiac damage has been attributed to SARS-CoV-2-related pathology contributing to increased risk of vascular events. Heart rate variability (HRV) is a parameter of functional neurocardiac integrity with low HRV constituting an independent predictor of cardiovascular mortality. Whether structural cardiac damage translates into neurocardiac dysfunction in patients infected with SARS-CoV-2 remains poorly understood. Hypothesized mechanisms of possible neurocardiac dysfunction in COVID-19 comprise direct systemic neuroinvasion of autonomic control centers, ascending virus propagation along cranial nerves and cardiac autonomic neuropathy. While the relationship between the autonomic nervous system and the cytokine cascade in general has been studied extensively, the interplay between the inflammatory response caused by SARS-CoV-2 and autonomic cardiovascular regulation remains largely unclear. We reviewed the current literature on the potential diagnostic and prognostic value of autonomic neurocardiac function assessment via analysis of HRV including time domain and spectral analysis techniques in patients with COVID-19. Furthermore, we discuss potential therapeutic targets of modulating neurocardiac function in this high-risk population including HRV biofeedback and the impact of long COVID on HRV as well as the approaches of clinical management. These topics might be of particular interest with respect to multimodal pandemic preparedness concepts.

## 1. Introduction

In December of 2019, the world saw the emergence of a novel coronavirus, the severe acute respiratory syndrome coronavirus 2 (SARS-CoV-2), leading to a new coronavirus disease, named coronavirus disease 2019 (COVID-19) [1]. The disease spread rapidly throughout China and then to the rest of the world, being officially declared a pandemic by the World Health Association (WHO) on 11 March 2020 [2]. As the pandemic progressed, it became clear that patients with a specific profile of preexisting conditions such as obesity, hypertension, diabetes, or smoking history had a higher risk of developing worse or fatal outcomes [3]. Similar to severe acute respiratory syndrome (SARS), COVID-19 is not only restricted to the airways but also is a systemic disease, which affects many organs including the heart [4,5,6,7,8,9].

In this narrative review, we are focusing on cardiac damage due to COVID-19 and its suspected pathophysiological mechanisms [10,11,12,13], as well as its effect on the autonomic function of the heart. Among possible mechanisms, viral entry into a cell via its interference with the renin–angiotensin–aldosterone system through binding of the viral protein S and the cellular angiotensin-converting enzyme 2 (ACE2) has been discussed [14,15]. This interaction causes a substantial down-regulation of cellular ACE2, which is attached to cellular membranes in multiple organs including the heart [4,16]. This down-regulation may compromise functional integrity of the cardiovascular system and may contribute to the worsening of outcomes [4,17].

Besides structural alterations, several studies suggested that SARS-CoV-2 infection impairs multiple functions of the autonomic nervous system [18,19,20,21,22,23,24,25,26,27,28]. It has been proposed that autonomic dysfunction in turn may contribute to induction of a pro-inflammatory cytokine storm in COVID-19, which is successively associated with a poor clinical prognosis [18,29,30,31]. The proposed underlying pathophysiological mechanism is a failure of the vagally mediated cholinergic anti-inflammatory pathway (CAP). This constitutes a negative feedback loop whereby the parasympathetic system down-regulates the inflammatory response back to homeostasis [32,33].

While diagnostic techniques such as the tilt table test or the Valsalva maneuver can quantitatively assess autonomic dysfunction of the cardiovascular system, heart rate variability (HRV) analysis allows for specific evaluation of neurocardiac autonomic function [34]. The role of HRV analysis as an easy-to-perform non-invasive marker of cardiovascular health and risk of mortality estimator has been well established in clinical practice and research [35,36,37]. However, the possible utility of HRV analysis as a biomarker for neurocardiac involvement and clinical prognosis in COVID-19 remains poorly understood [20,21,22].

Here, we reviewed the current scientific literature on neurocardiac function assessment via HRV in patients with COVID-19 to provide an overview of this potentially relevant research gap. In order to optimize the practical value of this review, we also included a brief summary of the principles of HRV analysis.

## 2. Search Strategy

We searched the database Medline through the PubMed interface using the following combinations of Medical Subject Headings and Boolean operators: ((“SARS-CoV-2”[MeSH Terms] OR “SARS-CoV-2”[All Fields] OR “COVID”[All Fields] OR “COVID-19”[MeSH Terms] OR “COVID-19”[All Fields]) AND ((“heart rate”[MeSH Terms] OR (“heart”[All Fields] AND “rate”[All Fields]) OR “heart rate”[All Fields]) AND (“variabilities”[All Fields] OR “variability”[All Fields] OR “variable”[All Fields] OR “variable s”[All Fields] OR “variables”[All Fields] OR “variably”[All Fields]))) OR (“autonomic nervous system”[MeSH Terms] OR (“autonomic”[All Fields] AND “nervous”[All Fields] AND “system”[All Fields]) OR “autonomic nervous system”[All Fields] OR “autonomic”[All Fields] OR “autonomical”[All Fields] OR “autonomically”[All Fields] OR “autonomics”[All Fields]) OR ((“autonomic nervous system”[MeSH Terms] OR (“autonomic”[All Fields] AND “nervous”[All Fields] AND “system”[All Fields]) OR “autonomic nervous system”[All Fields] OR “autonomic”[All Fields] OR “autonomical”[All Fields] OR “autonomically”[All Fields] OR “autonomics”[All Fields]) AND (“response”[All Fields] OR “responses”[All Fields] OR “respon-sive”[All Fields] OR “responsiveness”[All Fields] OR “responsivenesses”[All Fields] OR “responsives”[All Fields] OR “responsivities”[All Fields] OR “responsivity”[All Fields])) OR ((“cardiacs”[All Fields] OR “heart”[MeSH Terms] OR “heart”[All Fields] OR “cardiac”[All Fields]) AND (“damage”[All Fields] OR “damaged”[All Fields] OR “damages”[All Fields] OR “damaging”[All Fields])) OR (“autono-mous”[All Fields] OR “autonomously”[All Fields]) OR ((“heart”[MeSH Terms] OR “heart”[All Fields] OR “hearts”[All Fields] OR “heart s”[All Fields]) AND (“damage”[All Fields] OR “damaged”[All Fields] OR “damages”[All Fields] OR “damaging”[All Fields])). The identified articles were screened for titles and abstracts by authors L.S. and T.S., and eligibility of papers for inclusion was based on consensus. Since this is a narrative review, no data synthesis or study quality assessment was undertaken.

## 3. Heart Rate Variability

### 3.1. Physiological Principles

The human heart is capable of functioning autonomously without the need of an external stimulus because of the sinus node, which consists of specialized cells with pace-making properties [38]. Through the autonomic nervous system, the heart rhythm can be regulated according to physiological needs. The parasympathetic branch of the cardiac autonomic nervous system comprises myelinated nerve fibers from the ambiguous nucleus in the brainstem, having antiarrhythmic and bradycardic properties [39,40,41,42]. The sympathetic nervous system, on the other hand, opposes the parasympathetic activity by increasing the heart rate; its terminal neurons originate in the intermediolateral column of gray matter in the spinal cord [43]. The variability of the heart rate depends on the capability of cardiac pacemaker cells to respond to changes in sympathetic and parasympathetic tone, making HRV a measure of autonomic neurocardiac function.

### 3.2. Clinical Assessment

**Figure 1:**
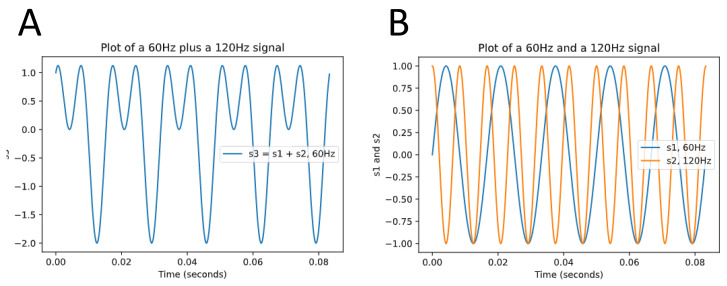
Decomposing a wave function into simple sinusoidal functions. (**A**) is a wave function s3 that consists of the sum of two simple sinusoidal functions, s1 (blue) and s2 (yellow) in (**B**). The complex signal composed of a myriad of frequencies is transformed by a filter into simple waves. Analysis of HRV using frequency domain methods functions in a similar fashion; the oscillation of the HRV is converted into a complex signal (analog to (**A**)), which then can be decomposed into various frequencies (analog to (**B**)) so that their individual contribution can be measured.

Assessment of HRV can be performed via time domain or frequency domain analysis [34]. Time domain analysis quantifies the variability of RR Intervals on electrocardiogram (ECG) to compute HRV, whereas frequency domain analysis of the ECG signal also referred to as spectral analysis is based on the conversion of RR Intervals into higher-order wave-shaped functions that reflect the oscillatory variability of the RR Intervals. These frequency functions are then decomposed into sinusoidal functions with varying degrees of contribution to the original curve [44] (Figure 1).

#### 3.2.1. Time Domain Analysis

Through computational algorithms, the R waves can be detected, and the RR Interval (also referred to NN Interval after correcting for artefacts) can be accurately measured using the beat-to-beat time recorder. With this data, parameters of HRV can be computed, the most widely used ones being listed below [34]:

- SDNN (milliseconds): Standard deviation of the NN intervals. As the name implies, this is the square root of variance. Since the variance increases with longer recording times, this value is highly dependent of the duration of measurement, which means that SDNN can only be used to compare HRV if both recordings lasted the same amount of time. The most commonly used recording settings are 5 min resting ECG and 24 h Holter ECG.
- SDANN (milliseconds): The recording is divided in 5 min segments, and the average of NN intervals for each segment is calculated. SDANN is the standard deviation of those averages.
- SDNN index (milliseconds): The recording is divided in 5 min segments, and for each segment, the standard deviation of all NN intervals is calculated. SDNN index is the mean of those standard deviations.
- RMSSD (milliseconds): Root mean square of successive differences. Here, each NN interval is subtracted from its neighbor, and the result is squared to yield only positive values. A mean of those values is calculated, and the RMSSD is the square root of this mean.
- NN50 (no units, natural number): the number of subsequent NN pairs whose difference is greater than 50 milliseconds.
- pNN50 (%): The value of NN50 divided by the total number of NN intervals.
- Geometric methods: The series of NN intervals are plotted as a geometric pattern, and the variability is analyzed using mathematical formulae based on the graphical or geometric traits.

#### 3.2.2. Frequency Domain Analysis

The heart rate can oscillate with varying frequencies. There are four main frequency ranges used for interpreting HRV: high frequency (HF) (0.15–0.4 Hz), low frequency (LF) (0.04–0.15 Hz), very low frequency (VLF) (0.003–0.04 Hz), and ultra-low frequency (ULF) (<0.003 Hz) [34]. The HF component usually represents efferent vagal activity, whereas the LF does not reliably represent any specific component of the autonomic nervous system but may be influenced by both sympathetic and parasympathetic activity levels [45]. Interpretation of very low frequency (VLF) and ultra-low frequency (ULF) bands is challenging as those cannot be attributed to specific autonomic systems.

The HF is highly correlated with time domain parameters RMSSD, NN50, and pNN50 as these depict short-term variation. When calculated based on a recording of 24 h, SDNN correlates with both HF and LF variations.

### 3.3. HRV as Biomarker of Cardiovascular Health

Assessment of HRV has been suggested to provide a valid biomarker of cardiovascular health [35,36,37,46,47]. Notwithstanding, its clinical usefulness is largely limited to the prediction of risk of death or arrhythmic events after myocardial infarction and the evaluation of diabetic and acute autonomic neuropathies [34,43]. However, its low technical demands and robust correlation with cardiac and overall health makes it a promising biological marker as measuring techniques and knowledge about its applications evolve.

### 3.4. Baroreflex Sensitivity

Changes to baroreflex sensitivity can contribute to a decrease in parasympathetic outflow while elevating sympathetic activity. This shift of autonomic balance, especially when developing chronically, may lead to cardiovascular dysregulation and disease [48]. Therefore, assessment of baroreflex sensitivity complements HRV as measure of cardiovascular autonomic integrity, contributing to risk analysis in patients with disorders of the cardiovascular system. The most established techniques to assess baroreflex sensitivity encompass the use of phenylephrine or other vasoactive adrenergic compounds, the Valsalva maneuver to enhance intrathoracic and intraabdominal pressure via straining as well as the application of a negative or positive pressure to the neck region to stimulate or deactivate baroreceptors. Baroreflex sensitivity can be assessed by analyzing spontaneous oscillations of systolic arterial pressure and RR intervals. This can be achieved by either logistic regression or spectral analysis of the association between specific oscillatory components of the two signals [49].

## 4. COVID-19 and Cardiac Damage

It is well-established that an infection with SARS-CoV-2 can lead to heart damage [4,5,6,7,8,9]. Moreover, it has been demonstrated in several studies that pre-existing cardiovascular and cerebrovascular morbidity worsens clinical outcomes in patients with COVID-19 [3,50,51,52,53]. The possible pathophysiological mechanisms whereby SARS-CoV-2 may affect the heart have been discussed extensively but due to heterogeneity in basic and clinical research studies the exact pathways have not yet been fully elucidated.

The way in which the host cells are infected by SARS-CoV-2 is through the binding of the viral protein S on its surface with the host cell’s angiotensin-converting enzyme 2 (ACE2) [54]. One of the consequences of this interplay is the down-regulation of ACE2 [4], limiting the organism’s capacity to convert the vasoconstrictor angiotensin 2 into the vasorelaxant angiotensin. The renin–angiotensin–aldosterone system (RAAS) plays a major role in the homeostasis and is expressed in multiple tissues including the heart [5,55,56]. The importance of ACE2 to the integrity of the cardiovascular system is underscored by the observation that its absence leads to ventricular dysfunction in animal models [4,17].

There is evidence of direct myocardial damage in pathological examinations of patients with COVID-19 [6,7]. Moreover, research suggested that an increased risk of myocardial infarction and heart failure in COVID-19 patients may be caused by cytokine storm with consequential vascular inflammation particularly involving TNF-α, interleukin-1β, and IL-6, plaque instability, and myocardial inflammation [57,58]. Some of the cytokines involved in this hyper-inflammation have a role inhibiting membrane channels of cardiomyocytes causing a channelopathy that predisposes to QT-prolongation related arrhythmias, which for instance contributes to multi-organ dysfunction [31]. It is also possible that indirect damage occurs as a consequence of infection, sepsis, disseminated intravascular coagulopathy, hypoxia, and/or stress-induced cardiomyopathy [10].

## 5. Neurocardiac Autonomic Dysfunction in COVID-19

To date, literature linking heart rate variability and COVID-19 is scarce. Out of 403 articles that were identified on screening of titles and abstracts, only three investigated the relation between HRV and COVID-19. In a small observational study at an intensive care unit of Mount Sinai Medical Center, 17 patients were enrolled. In the timespan of 7 days, a small wearable device recorded a one-lead ECG for 7 min in the morning, and HRV was calculated using SDNN; the levels of C-reactive protein (CRP) were also measured daily. A reduction in the HRV correlated with an increase of CRP of more than 50% in a time-window of up to 72 h. The sensitivity of the HRV decrease was 83.3%, the specificity was 75%, and the positive and negative predictive values were 90.9% and 60%. However, these changes of HRV did not correlate with clinical outcomes. Limitations of this study are the small sample size, the lack of a control group, and the absence of standardized frequency of laboratory testing and therapeutic interventions [20].

The results of a case–control study also suggested that a SARS-CoV-2 infection may cause neurocardiac autonomic dysfunction. In this observational investigation, 63 patients with a positive polymerase chain reaction (PCR) test for COVID-19 were matched with 43 healthy controls by age and gender. The HRV was measured in an ambulatory setting using an ECG system in lead II for 5 min under standardized conditions. Assessment of HRV was carried out by computing SDNN and RMSSD. Patients with conditions that could influence the HRV, such as intake of beta-blockers, inhaled or oral beta-mimetics, history of cerebrovascular accident, coronary artery disease, among others were excluded. Additionally, patients with severe COVID-19 requiring oxygen or intensive care were excluded. The authors used a cutoff of ≥40 ms for the RMSSD as a sign of increased parasympathetic tone based on the results of a systematic review of normative HRV values in healthy populations [21,59]. The authors observed that 30.6% of the subjects with COVID-19 had an increased parasympathetic activity measured via RMSSD. In comparison, in a healthy cohort, 4.7% of subjects displayed an RMSSD ≥ 40 ms. The authors concluded that patients with a SARS-CoV-2 infection have an elevated parasympathetic tone. While it seems reasonable to assume that patients with COVID-19 display autonomic imbalance, it remains to be answered why parasympathetic activation is not paralleled by an equal or even more pronounced sympathetic stress response resulting from acute systemic inflammation. Noteworthy, frequency domain analysis showed a reduced parasympathetic activity in patients with COVID-19 in this study, thus contradicting the results from the time domain analysis.

Another observational study, which recruited sixteen COVID-19 positive patients from a single intensive unit of the Mostoles General University Hospital in Madrid, aimed to establish the HRV as a prognostic factor in sixteen SARS-CoV-2 infected patients. The HRV was measured using the analgesia nociception index monitor (ANI monitor) that calculates the normalized unit spectral indices of HF as the ratio between the absolute value of the HF and the SDNN (also referred to as HFnu), providing a value between 0 and 100 (also referred to as ANI). This number ultimately represents an estimate of the ratio between the parasympathetic tone and the activity of different spectral components. The authors measured the following parameters daily in the morning during four minutes: ANIm (mean ANI for the measured time), ANIi (instant ANI for the past 120 s), and the SDNN (also referred as “energy” in this study). To minimize external influences, caregivers were instructed to avoid changes in drug rates, patient mobilization, and invasive procedures before measurements. Furthermore, they assessed the Richmond Agitation–Sedation scale (RASS), estimated the clinical severity using the SOFA (Sequential Organ Failure Assessment) score, and assessed blood levels of IL-6, CRP, and procalcitonin at day one. After 30 days, the medical record of the patients was reviewed, and outcomes were extracted. Two patients were transferred and could not be included in the final analysis, seven survived, and seven deceased. The main findings comprised higher ANIm figures in the deceased group indicating an increased parasympathetic tone. SDNN correlated inversely with the SOFA score, but not with mortality or with inflammatory markers. Furthermore, a threshold of 80 for the ANIm value had a sensitivity of 100%, a specificity of 85.7%, a positive predictive value (PPV) of 87.5% and a negative predictive value (NPV) of 100% for mortality. A subgroup analysis in patients with RASS-4/-5 was undertaken to filter out the mutual influence between ANI monitor values, drug dosage, and vigilance, since homogeneity of such parameters was higher in this specific cohort. This filter significantly boosted the capacity to predict mortality of the ANIm (sensitivity, specificity, PPV, and NPV; all were 100%) as well as of the SDNN (sensitivity of 71.4%, specificity of 75%, PPV of 83.3, and NPV of 60%). However, the study was limited by the small sample size and its monocentric design as well as the possible influence of analgesic and sedation drugs, neuromuscular blockade, and the use of immune-modulating drug like monoclonal antibodies and glucocorticoids [22]. The main finding of this article showing a worse prognosis without evidence of increased inflammation markers is contrasting the aforementioned observational study from Mount Sinai medical center, although both compared patients being treated in an ICU.

To better understand the relation between HRV and COVID, it may be worthwhile to look at other autonomic functions outside the heart. A retrospective study at the Mayo Clinic identified 27 Patients with symptoms of para-/post-infectious autonomic dysfunction after SARS-CoV-2 infection. Of them, 17 had abnormalities on peripheral autonomic functions consistent with autonomic neuropathy [23]. An observational study in an Indian population identified 13 patients with sudomotor dysfunction out of 50 with unspecific long-lasting symptoms after COVID-19 [24]. A report of two pediatric German patients comprised one case of autonomic dysfunction with elevated vagus activity in acute COVID-19 and another case of orthostatic hypotension two weeks after cessation of the acute infection [25]. Furthermore, a case series of six patients reported dysautonomia in patients with autonomic symptoms after remission of acute COVID-19 symptoms, ranging from orthostatic hypotension and postural tachycardia to cardiovagal impairment [26]. Similarly, a retrospective analysis in Chile identified 20 patients with signs of orthostatic dysautonomia on a tilt table or 10 min standing test [28]. A, report presented a case of dysautonomic signs and symptoms (sinus arrhythmia, postural hypotension, intermittent profuse sweating, constipation, erectile dysfunctions, and squeezing sensation in the chest) during an acute SARS-CoV-2 infection preceding an acute axonal motor neuropathy [27]. Lastly, prolonged bed rest with subsequent muscle weakness as well as development of critical illness polyneuropathy and critical illness myopathy might contribute to dysautonomia through feedback loops as well as direct damage to autonomic nerve fibers [60]. Early initiation of multidisciplinary rehabilitation after ICU stay might be an approach to counteract COVID-19-related dysautonomia, which could also be relevant to future pandemic preparedness strategies.

### 5.1. Possible Pathophysiological Mechanisms of Autonomic Neurocardiac Dysfunction in Patients with COVID-19

**Figure 2:**
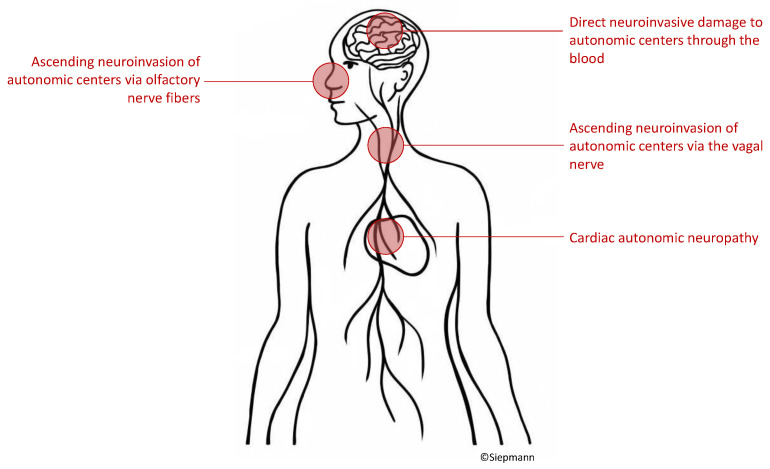
Possible pathways whereby SARS-CoV-2 might reach neural targets within the autonomic nervous system to cause neurocardiac dysfunction.

To date, indisputable evidence on how COVID-19 leads to autonomic nervous system damage is lacking. Hypothesized mechanisms include virus ascent through cranial nerves [18,61], hematogenous infiltration of autonomic centers [62,63,64], or cardiac autonomic neuropathy [23,26,65] as depicted in Figure 2.

#### 5.1.1. Virus Ascent via Cranial Nerves

Even though the primary infection site of SARS-CoV-2 are the airways, early reports showed that a secondary central neurological affliction is possible [66], with later works histologically corroborating these findings [67]. A study found evidence of ACE2 receptors in olfactory mucosa and indicated possible subsequent invasion of other neuroanatomical structures such as the brain stem [61]. Based on observations, the invasion from autonomic structures in the brain stem might seem reasonable. However, another study contradicted this hypothesis by demonstrating that ACE2 expression is not present in olfactory neurons but solely on other cells of the olfactory mucosa [68,69]. Importantly and in favor of the first of the two hypotheses, an immunohistochemical mapping of the murine brain showed that ACE2 expression is highest in the dorsal motor nucleus of the vagus, area postrema, and the nucleus tractus solitarius (NTS) [70,71,72] and all parts of the dorsal vagal complex and important autonomic nuclei [18,73,74].

#### 5.1.2. Hematogenous Invasion of the Central Nervous System

The brain is under normal circumstances well protected from infectious agents by the blood–brain barrier, a complex layer of mechanisms that hinders most external aggressions to the central nervous system. Certain pathogens, however, are able to overcome the blood–brain barrier to infect nervous cells and some authors have discussed this mechanism as one of the possible pathways towards neuroinvasion of SARS-CoV-2 [61,62,63,64]. The first step in this scenario would be a viremia occurring through the invasion of the endothelium in the lungs. From the circulation the virus could then enter the nervous system either through the neuronal endothelium or using leukocytes as vector.

#### 5.1.3. Cardiac Autonomic Neuropathy

A SARS-CoV-2 medicated autonomic neuropathy could also explain cardiac autonomic dysfunction. There is a lack of original research investigating this aspect, but in theory, possible mechanisms could comprise direct viral damage to autonomic nerve fibers or secondary damage following an immune response [23,26,64,65]. Moreover, the peripheral autonomic nervous system is knowingly susceptible to acute autoimmune mediated demyelization [75,76]. However, due to absent comprehensive original research on this hypothesis, this potential mechanism remains purely speculative. When considering the possibility of cardiac autonomic neuropathy related to COVID-19, it needs to be acknowledged that a considerable amount of structural heart damage seems to be associated with the virus infection. Mechanisms of cardiac damage due to infection with SARS-CoV-2 might be due to direct damage mediated by invasion of cardiomyocytes by SARS-CoV-2 that infects via a cathepsin and angiotensin-converting enzyme 2 (ACE2), inflammatory, and thrombotic injury to endothelial cells. Moreover, indirect pathophysiological mechanism might contribute to SARS-CoV-2-related cardiac damage. Proposed pathways of indirect damage comprise elevated myocardial demand due to tachycardia and hypotension induced by sepsis or hypoxemia as well as impairment of cardiac functional integrity due to arrhythmia, ischemia, coronary thrombosis, or stress-induced cardiomyopathy, which is also referred to as Takotsubo syndrome [77].

## 6. Crosstalk of the Immune System and the Autonomic Nervous System in the Context of COVID-19

Another important reason to look closely at the autonomic nervous system function in the pathophysiology of COVID-19 is constituted by its possible role in the dysregulated immune reaction also known as cytokine storm. To comprehend this interaction, it may be useful to consider the temporal course of the SARS-CoV-2 infection. The beginning of the disease is a typical acute infection of the airways after an initial incubation period with symptoms that may include dyspnea, cough, nausea, fever, fatigue, ageusia, and anosmia, triggering an innate immune response [78,79]. The pulmonary invasion is accompanied by tissue damage and facilitation of the inflammatory process and its consequences such as vasodilatation, increased endothelial permeability, and recruitment of leukocytes, which worsens the damage to the lungs [79]. Patients often present with low oxygen saturation–often severe–without tachypnea or dyspnea, also labeled “happy hypoxemia”. A possible explanation for this phenomenon may be inflammation of the nucleus tractus solitaries, which plays a crucial role in the regulation of respiratory function [80].

About a tenth of the patients enter a delayed inflammatory phase in which the inflammatory reaction progresses systemically [79], sometimes despite reduction of viral load, causing along with it coagulopathy [81], cardio-respiratory dysregulation [50], and organ failure. Most patients susceptible to worse outcomes have preexisting conditions that have already established chronic autonomic dysregulation such as hypertension, diabetes, chronic kidney disease, and heart failure [29,30]. This dysfunction can be either extrinsic due to a sympathetic overtone and homeostatic compensation from unimpaired organs or intrinsic as a consequence of direct damage (for example, due to diabetes) [82].

The vagus nerve plays an important role in the regulation of the inflammatory response through the cholinergic anti-inflammatory pathway, first described by Borovikova and Tracey et al. [32,33]. Afferent inflammatory impulses are processed in the dorsal vagal complex, generating an efferent response in a somatotopic fashion at the injury site with acetylcholine secretion, which down-regulates the transcription of pro-inflammatory cytokines back to homeostasis.

The late phase of COVID-19, also known as “cytokine storm”, is a vicious cycle of inflammation that occurs due a lack of counter-regulation [83]. It is thus possible that a combination of factors including a preexisting autonomic dysfunction due to comorbidities with additional direct or indirect viral damage may play a role in this severe disease stage.

## 7. Cardiovascular Dysautonomia beyond the Acute Phases of COVID-19

Research showed that autonomic dysfunction due to COVID-19 is linked to both acute and sustained complications and may contribute to the symptoms attributed to long COVID syndrome [84]. In fact, a recent meta-analysis was able to identify more than 50 long-term symptoms related to a history of COVID-19, and the majority of which are related to dysfunction of the autonomic nervous system. Ref. [85] Long COVID still seems to be a problem with increasing numbers around the globe with meta-analytic evidence on an estimated pooled prevalence of 0.43 (95% confidence interval: 0.39–0.46) [86]. A recent systematic review and meta-analysis synthesized data from studies that reported symptoms of autonomic dysfunction during the acute phases of COVID-19 as well as during the long COVID phase [87]. The authors found that cardiovascular dysfunction due to impairment of the autonomic nervous system are frequent in both the acute and long COVID-19 stages, but the spectrum of clinical manifestation differs between both phases of the disease. The acute phases of COVID-19 frequently present with reflex syncope and blood pressure instability possibly related to a pro-inflammatory condition with hypovolemia caused by fever or cytokine-evoked vasodilation with an inverse correlation of HRV with pro-inflammatory markers. Interestingly, cardiovascular autonomic dysfunction appears to show higher rates of recovery in patients with acute COVID-19 than in those with long COVID, indicating that persistent dysautonomia may parallel other symptoms of long COVID such as fatigue and mood swings [87,88,89]. Moreover, patients with cardiovascular autonomic dysfunction in acute COVID-19 phases seem to younger and more often female than those with cardiovascular autonomic dysfunction linked to long COVID further supporting different pathophysiologies underlying both conditions [87]. While postural orthostatic tachycardia occurs in up to 62% of patients with long COVID, the condition is less frequently seen in patients with acute COVID-19. However, multiple autonomic disturbances might occur outside the cardiovascular system, e.g., in the sudomotor, gastrointestinal, or genital system, underscoring the need for comprehensive observational research on dysautonomia in acute and long COVID conditions [87,90].

Differences in the clinical manifestation of dysautonomia in patients during acute or long COVID phases may be related to differences in the underlying pathophysiological mechanism. The molecular mechanisms of the long COVID syndrome are not fully elucidated and are likely multi-faceted comprising sequels of direct structural damage to target organs mediated by virus invasion during the early infection phases as well as sustained consequences of systemic inflammation and cytokine storm [91,92]. Although the impairment of structures of the autonomic nervous system has not yet been fully elucidated, neither for acute nor chronic phases, cumulative research indicates a chronic auto-immune component to the pathophysiology of long COVID mediated by autoantibodies that are directed against various epitopes including different receptors and glycoproteins expressed on cellular membranes [93,94,95,96]. Additional possible mechanisms whereby long COVID may lead to dysautonomia include direct virus invasion of the medulla and/or hypothalamus through the blood or neural pathways, chronic elevation of sympathetic tone by persistent inflammation and hypoxia as well as chronic imbalance of the renin–angiotensin system [96]. The diagnostic value of HRV assessment in the context of cardiovascular dysautonomia linked to long COVID remains uncertain. However, a prospective study in 40 participants with previous COVID-19 discharged for six months showed that HRV differed between patients with and without persistent diffusion dysfunction and had a tight association with pulmonary fibrosis. The capacity of HRV analysis to predict cardiovascular long-term outcomes in patients with long COVID remains to be determined.

Along these lines, it needs to be acknowledged that there is a substantial heterogeneity in results with respect to the impact of long COVID on specific parameters or HRV. For instance, an observational study found that SDNN and HF were decreased in long COVID patients compared to controls while RMSDD and LF displayed no between-group difference [97]. These observations, viewed in conjunction with an additionally detected increase in LF/HF ration, would be consistent with a long COVID-related attenuation of parasympathetic activity. However, in another study, a subgroup of patients with long COVID symptoms persisting over more than 3 months displayed increased SDNN and decreased RMSDD compared to patients without SARS-CoV-2 infection while LF, HF, and LF/HF ratio remained unaltered [98]. In this analysis, the reduced RMSDD might be consistent with predominantly parasympathetic dysautonomia, but the elevation of SDNN remained difficult to explain. While these discrepancies in the literature might be partially explained by methodological heterogeneity, further real-world and big data analyses might be helpful to identify patterns of cardiovascular dysautonomia in long COVID patients more precisely.

## 8. HRV Biofeedback and Its Potential to Modify Cardiovascular Outcomes in Patients with COVID-19

### 8.1. Background

**Figure 3:**
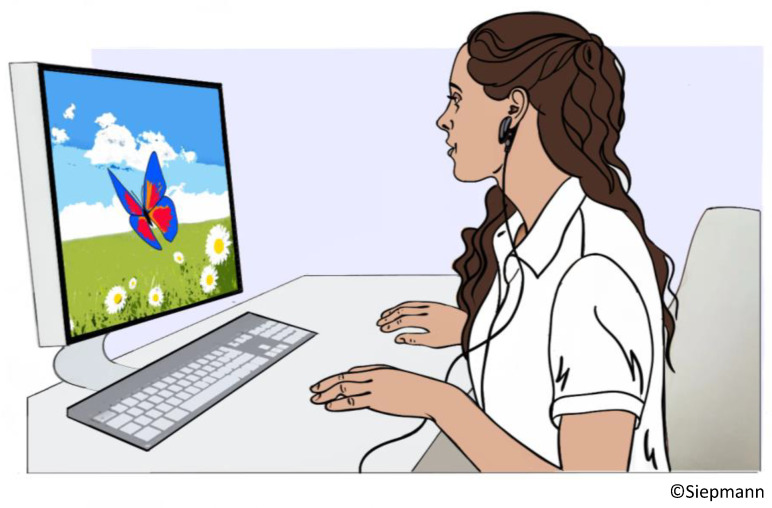
HRV Biofeedback training. The drawing shows a subject undergoing HRV biofeedback with continuous measurement of HRV via an ear clip and visualization on the computer screen via a hovering butterfly that rises in response to an increase and descents during a decrease in HRV while the subjects perform a metronomic breathing exercise to increase parasympathetic outflow and thereby HRV.

Biofeedback targeting HRV was designed to increase vagal tone via metronomic breathing and thereby improve cardiac autonomic function and clinical outcomes in patients with disorders that are associated with cardiovascular dysautonomia and elevated sympathetic activity [99]. The biofeedback training comprises breathing at a specific resonant frequency that varies for each individual (but usually falls within the range from 4.5–6.5 respiratory cycles per minute). This breathing pattern stimulates the baroreflex and results in a reflectory vagal stimulation that intensifies the respiratory sinus arrythmia leading to elevated parasympathetic activity. The actual biosignal feedback loop is based on visualizing HRV on a computer screen in real-time. The patients watches the screen while exercising the metronomic breathing. A levitating object, e.g., a butterfly, represents HRV. A rising butterfly indicates an increase in HRV while descent indicates decrease in HRV. The instructed breathing frequency is also visualized by a moving bar that indicated when to breath in and out. The procedure is depicted in Figure 3.

This biobehavioral treatment has been applied to neuropsychiatric and cardiovascular disorders in several research studies with overall beneficial effects on cardiac autonomic function and in some cases on disease-specific clinical outcomes, but large confirmatory efficacy trials are lacking to date [100,101,102,103,104,105,106,107,108,109,110,111].

### 8.2. HRV Biofeedback in Cardiovascular and Cerebrovascular Disease

HRV biofeedback has been studied as a non-pharmacological, low-risk therapy for an increasing number of cardiovascular disorders. For instance, a randomized trial published by Del Pozo and colleagues investigated whether biofeedback treatment could increase HRV in patients with documented coronary artery disease. A significant increase in SDNN was observed in patients with coronary artery disease following six weeks of biofeedback training and compared to control subjects undergoing conventional therapy. This effect was found to be sustained even after 18 weeks [101]. Another study demonstrated that HRV biofeedback increases decreases blood pressure in prehypertensive subjects by modulating autonomic cardiovascular function, thereby demonstrating a potentially beneficial effect of the intervention on a disease-related clinical outcome beyond modulation of HRV [98]. Another randomized controlled trial tested a portable, battery-powered device to provide small sessions of HRV biofeedback after an initial introductory session with a certified expert in patients with a history of myocardial infarction. This study supported feasibility of self-guided HRV biofeedback in the vast majority of participants and demonstrated an increase in HRV in the population at risk [104]. In three studies, an increase in HRV following HRV biofeedback was associated with improved functional outcome. A randomized interventional study tested the effect of a 6-week HRV biofeedback treatment in patients with heart failure ranging from NYHA I to III. This trial was able to show an improved exercise tolerance per 6 min walk test following HRV biofeedback [105]. A randomized sham-controlled trial with patients in patients undergoing acute multidisciplinary stroke unit care in Germany also showed elevated HRV and alleviated symptoms related to autonomic disturbances three months after the intervention [100]. Similar observations were made in a Taiwanese population of stroke survivors that displayed improved HRV and alleviated symptoms of anxiety and depression after HRV biofeedback [106]. A small randomized control trial with a wait-list control group design was able to demonstrate that HRV biofeedback reduced reactivity of the autonomic nervous system during anger events and enhanced recovery reactivity of the autonomic nervous system after anger events in patients with coronary artery disease [107]. To approach long-term effects of HRV biofeedback, a randomized study in 210 patients with coronary artery disease compared hospital clinical outcomes one year after the intervention and found improved cardiovascular prognosis as well as a beneficial modulation of neurocardiac autonomic homeostasis and baroreflex sensitivity [108]. In fact, patients in the HRV biofeedback group displayed fewer all-cause readmissions as well as all-cause emergency visits. Due to the lack of deaths in both groups, it was not possible to analyze mortality. Lastly, a systematic review of the influence of HRV modulation on cardiovascular outcomes was able to include 12 studies into the final analysis, ultimately concluding that HRV biofeedback may have positive therapeutic effects on clinical outcomes related to several cardiovascular diseases but remained unable to draw any conclusion on the intervention’s effect on cardiovascular mortality [99]. To date, large confirmatory phase 3 randomized controlled trials to confirm efficacy of HRV biofeedback in improving clinical outcomes in cardiovascular disease are lacking.

### 8.3. Possible Implictaions of HRV Biofeedback in Patients with COVID-19

The idea of applying HRV biofeedback in patients who are suffering from COVID-19 in order to modulate autonomic hemostasis in these patients at risk was first raised not even a year after the outbreak of the pandemic. Peláez-Hernández and colleagues published a short position paper, which included a narrative review of the current body of evidence. They concluded that COVID-19 survivors display autonomic dysfunction related to cardiovascular impairment that may lead to poor clinical outcomes and mutual additive deterioration in conjunction with psychological sequelae of the disease [109]. Furthermore, they speculated that HRV biofeedback training might offer a valid technique to counteract this possible vicious cycle. Another postulated approach to counteract the consequences of infection with SARS-CoV-2 using HRV biofeedback comprises its use in patients with long COVID. Long COVID, also referred to as post-COVID-19 syndrome, is defined as sustained symptoms that persist at least 12 weeks following COVID-19 [110]. About one out of seven patients with COVID-19 develops long COVID and may display a broad spectrum of symptoms including fatigue, breathing problems, cardiac arrhythmia or palpitations, vertigo, and brain fog. Autonomic dysfunction is associated with many of these manifestations of long COVID. A currently ongoing study, the HEART rate variability biofeedback for long COVID symptoms (HEARTLOC) trials, is investigating whether HRV biofeedback might be able to modulate autonomic function in patients with long COVID, thereby alleviating symptoms related to dysautonomia [111]. The sample size (*n* = 30) is rather low, but if positive, the trial will form a basis for confirmatory follow-up research. Another ongoing trial is aiming to test the feasibility of HVR biofeedback in patients suffering from long COVID and additionally to gather data on the intervention’s effect on cognition, perception of pain, fatigue, symptoms of anxiety, and depression as well as quality of life [112]. This study will also be limited by a small sample of 20 patients but may add valuable insights into the feasibility of HRV biofeedback in patients with long COVID. This might be of particular importance since HRV biofeedback is based on metronomic breathing at a slow frequency that is known to increase parasympathetic tone but might be challenging for patients with persisting breathing difficulties.

## 9. Conclusions and Future Directions

The role of neurocardiac dysfunction in the course of COVID-19 as well as the capacity of HRV analysis to predict clinical outcome in affected patients are poorly understood because of a substantial lack of prospective research. The few studies available suggest some impairment of neurocardiac regulation in COVID-19, but the prognostic value with respect to clinical outcomes as well as its pathophysiological link to systemic inflammation remains to be elucidated. Several mechanisms underlying neurocardiac dysfunction in COVID-19 patients have been discussed, such as direct systemic neuroinvasion of autonomic control centers, ascending neural virus propagation, and cardiac neuropathy, but the existing data do not allow specific conclusions on this potentially important complication of infection with SARS-CoV-2. The mechanism whereby COVID-19 leads to neurocardiac dysfunction seems to differ substantially from the pathophysiology of dysautonomia linked to long COVID. Prospective research on neurocardiac function in large cohorts of COVID-19 and long COVID patients is urgently needed to improve our understanding of the disease and identify potential therapeutic targets. Since HRV can be modified non-pharmacologically using biofeedback based on metronomic breathing in cardiovascular disease, future research might also aim at demonstrating efficacy of HRV in counteracting dysautonomia in patients with COVID-19 and long COVID, and some smaller proof of concept studies being already underway. In summary, the value of HRV as diagnostic and therapeutic target in acute COVID-19 and in the long COVID condition is promising but remains poorly understood. To date, substantial evidence to support a definite diagnostic and therapeutic value of HRV in acute COVID-19 and in the long COVID condition is lacking. While our narrative literature review identified potentially helpful diagnostic and therapeutic targets of cardiovascular autonomic function in patients with COVID-19, interventional prospective research is needed to contribute to effective strategies of managing cardiovascular dysautonomia in the context of a pandemic preparedness.

## Data Availability

Not applicable.
